# Variations in the prevalence of point (pre)hypertension in a Nigerian school-going adolescent population living in a semi-urban and an urban area

**DOI:** 10.1186/1471-2431-10-13

**Published:** 2010-03-09

**Authors:** Chukwunonso ECC Ejike, Chidiebere E Ugwu, Lawrence US Ezeanyika

**Affiliations:** 1 Department of Biochemistry, Michael Okpara University of Agriculture, Umudike, Nigeria; 2 Department of Biochemistry, Kogi State University, Anyigba, Nigeria; 3 Department of Biochemistry, University of Nigeria, Nsukka, Nigeria

## Abstract

**Background:**

Hypertension has been shown to start in early life and to track into adulthood. Detecting adolescents with hypertension and prehypertension will aid early intervention and reduce morbidity and mortality from the disorders. This study reports the point-prevalence of the two disorders in a semi-urban and an urban population of school-going adolescents in Nigeria.

**Methods:**

A total of 843 adolescents from two places of domicile were studied. Their blood pressures and anthropometric indices were measured using standard protocol. Point-hypertension and point-prehypertension were defined with respect to each subject's gender, age and height. The prevalence of the disorders was calculated and reported age-wise and nutritional status-wise.

**Results:**

The prevalence of point-prehypertension in the semi-urban area was 22.2% (20.7% for girls and 23.1% for boys) while it was 25.0% (21.8% for girls and 29.2% for boys) in the urban area. The prevalence of point-hypertension was 4.6% (4.1% for girls and 4.8% for boys) in the semi-urban area and 17.5% (18.0% for girls and 16.9% for boys) in the urban area. Point-prehypertension was not detected among the thin subjects of both places of domicile. The prevalence of point-prehypertension was similar in both the urban and semi-urban areas among the subjects who had normal BMI-for-age, and over-weight/obese subjects respectively. From the semi-urban to the urban area, the prevalence of point-hypertension increased approximately 3-folds among thin and normal BMI-for-age subjects, and 10-folds among overweight/obese subjects. Systolic hypertension was more preponderant in both the semi-urban and urban areas.

**Conclusions:**

The prevalence of both disorders is considerably high in the studied populations. Urgent pediatric public health action is needed to address the situation.

## Background

High blood pressure causes one in every eight deaths world wide, making it the third leading killer in the world [1]. About one billion adults, the world over, had hypertension in the year 2000, and the number is expected to rise to 1.56 billion in 2025 [2]. Studies have shown that hypertension may begin in adolescence, perhaps even in childhood, and tracks into adulthood [3-5]. Hypertension in adolescents however often goes undiscovered because adolescents are generally healthy and visit a physician only when they are very ill. For this reason, the measurement of blood pressure even among adolescents has been recommended [6]. High blood pressure is the major risk factor for cardiovascular diseases and detecting it in adolescents may be vital to cardiovascular disease control in adult life [7]. Detecting adolescents with prehypertension would help in identifying those to be targeted for early management. Data from adolescent hypertension studies show prevalence rates ranging from 1.3% to 21.6%, [8-35] but data on the prevalence of prehypertension in adolescents is rather sparse. This study investigated the prevalence of point-prehypertension and point-hypertension in adolescents from two localities in Kogi state, Nigeria. The findings are expected to be useful in pediatric public health policy formulation and action.

## Methods

This study is an analysis of a sub-set of a study of adolescents in Kogi state, Nigeria [36]. The study site and methods were appropriately described in the said study. Data for 843 adolescents (attending public secondary schools) in Lokoja (urban) and Ajaokuta (semi-urban) who were between 13 years and 18 years were included for analysis in the present study. Those aged 10-12 years and 19-20 years, and rural dwellers, in the previous study were excluded because their sample sizes were so small that objective statistical deductions cannot be made from them. Semi-urban area, here, refers to a town where some of the inhabitants are skilled or unskilled artisans, but still engage in farming and the others, say 60%, engage in subsistence farming; while urban area refers to a town with virtually all the trappings of a city - good paved roads, electricity, pipe-borne water and almost all the inhabitants do not engage in subsistence agriculture.

Three separate blood pressure (BP) readings were taken per subject, at two minutes intervals, after an initial 10 minutes rest, in a seated position, using an automated digital monitor (Omron HEM-741 CINT), and appropriate cuff sizes. The device has an error of measurement of ± 3 mmHg, according to the manufacturers. The average of the last two readings was recorded for both systolic and diastolic blood pressures of each student. Each subject was thereafter asked if he/she ever had a BP measurement in the past, and the response recorded. The same trained personnel took all blood pressure measurements.

Height was measured (to the nearest 0.5 cm) using a non-elastic measuring tape, fastened to a vertical wall, with the student standing on bare feet. Weight was measured (with the student on bare feet and with light clothing) using an electronic weighing balance, to the nearest 0.1 kg. From the heights and weights got, Body Mass Index (BMI) was calculated using the formula BMI = Weight (kg)/[Height (m)]^2^. All the equipments were calibrated each morning according to the manufacturer's instructions. All anthropometric variables were measured by the same trained personnel.

Normal blood pressure (NBP) is taken as systolic and diastolic blood pressure that is < 90^th ^percentile for gender, age and height. Prehypertension is taken as systolic and diastolic blood pressure ≥ 90^th ^percentile, but < 95^th ^percentile for gender, age and height or ≥ 120/80 mmHg [6]. Hypertension is taken as systolic and diastolic blood pressure ≥ 95^th ^percentile for gender, age and height [6]. Subjects whose blood pressures were ≥ 90^th ^percentile for gender, age and height were taken to have elevated blood pressure (EBP). Thinness and overweight/obesity were defined as BMI-for-age < 5^th ^percentile and > 85^th ^percentile of the first US National Health and Nutrition Examination Survey (NHANES I) 1971-1974 reference data for blacks [37,38] as approved by the World Health Organization (WHO) [39]. Normal BMI-for-age was taken as values ≥ 5^th ^percentile but ≤ 85^th ^percentile of the reference data.

Only subjects who were apparently without any overt signs of ill health and who gave an informed verbal consent after consulting with their parents/guardians were allowed to participate in the study. The study protocol and design were approved by the Ethics committee of the Department of Biochemistry, Kogi state University, Anyigba.

Descriptive statistics was done for SBP and DBP for subjects with NBP and EBP within the ages and data presented in line graphs. Group comparisons were done between the sexes for SBP and DBP in all the ages using the ANOVA test and p values < 0.05 were considered significant. To calculate the prevalence of the disorders, we divided the number of such cases by the number of subjects in that category, and multiplied the answer by 100. All data analyses were done using the statistical software SPSS version 11.0 (SPSS Inc. Chicago IL). Data are presented in tables and line graphs.

## Results

The prevalence of point-prehypertension in the semi-urban population increased with increasing age from 15.1% (13 years) to 37.2% (18 years). For the semi-urban dwelling boys, point-prehypertension prevalence was highest at 17 years, while for their female counterparts, it was highest at 18 years. Irrespective of sex, the prevalence of point-prehypertension in the semi-urban area is 22.2% (Table 1).

**Table 1:** Age-wise prevalence of point-prehypertension and point-hypertension in the population

AGE (Yrs)	SEMI-URBAN AREA									URBAN AREA								
	
	BOYS			GIRLS			TOGETHER			BOYS			GIRLS			TOGETHER		
	
	N	PHT (%)	HT (%)	N	PHT (%)	HT (%)	N	PHT (%)	HT (%)	N	PHT (%)	HT (%)	N	PHT (%)	HT (%)	N	PHT (%)	HT (%)
13	65	16.9	1.5	41	12.2	2.4	106	15.1	1.9	3	0.0	0.0	16	6.3	0.0	19	5.3	0.0
14	86	17.4	8.1	46	26.1	4.3	132	20.5	6.8	10	10.0	40.0	33	18.2	6.1	43	16.3	14.0
15	58	20.7	6.9	36	19.4	5.6	94	20.2	6.4	36	13.9	13.9	48	27.1	18.8	84	21.4	16.7
16	30	30.0	3.3	28	32.1	7.1	58	31.0	5.2	35	34.3	17.1	58	22.4	22.4	93	26.9	20.4
17	27	40.7	3.7	23	21.7	4.3	50	32.0	4.0	30	36.7	6.7	33	18.2	27.3	63	27.0	17.5
18	24	37.5	0.0	19	36.8	0.0	43	37.2	0.0	40	40.0	22.5	18	33.3	22.2	58	37.9	22.4
Total	290	23.1	4.8	193	20.7	4.1	483	22.2	4.6	154	29.2	16.9	206	21.8	18.0	360	25.0	17.5

PHT and HT stand for point-prehypertension and point-hypertension respectively

In the urban area, the prevalence of point-prehypertension also increased with increasing age. At 13 years, the figure stood at 5.3% but increased more than 7-folds to 37.9% at 18 years. As much as 33.3% of the girls aged 18 years in the urban area had point-prehypertension. The prevalence of point-prehypertension in the urban area irrespective of sex is 25.0% (Table 1).

Point-hypertension was most prevalent in subjects aged 14 and 15 years (6.8% and 6.4% respectively) in the semi-urban area. In this place of domicile, point-hypertension was absent in those aged 18 years. A total of 4.6% of the semi-urban dwellers (4.8% for boys and 4.1% for girls) had point-hypertension (Table 1).

In the urban area, point-hypertension was absent in adolescents aged 13 years. The prevalence of point-hypertension was highest (22.4%) at age 18 years, irrespective of sex, in the urban area. However, it was more prevalent at 14 years for males (40.0%) and 17 years for females (27.3%). The prevalence of point-hypertension in the urban area is 16.9% (boys), 18.0% (girls) and 17.5% (irrespective of sex) (Table 1).

More than 80% of the studied population irrespective of place of domicile had normal BMI-for-age values. In the semi-urban and urban areas, 25.3% and 26.2% respectively of those with normal BMI-for-age had point-prehypertension, while 4.6% and 15.0% of the same group, irrespective of sex had point-hypertension. Urban-dwelling boys who had normal BMI-for-age had a higher prevalence of point-prehypertension than their semi-urban counterparts (31.9% and 28.4% respectively). Those of the females were however closer (21.9% for urban females and 21.5% for semi-urban females). The prevalence of point-hypertension was close between urban-dwelling males and females who had normal BMI-for-age (15.2% and 14.8% respectively) but not so in the semi-urban population (5.6% for males and 3.4% for females) (Table 2).

**Table 2:** BMI-wise prevalence of point-prehypertension and point-hypertension in the population

	SEMI-URBAN AREA									URBAN AREA								
	
	BOYS			GIRLS			TOGETHER			BOYS			GIRLS			TOGETHER		
	
Nutritional status	N	PHT (%)	HT (%)	N	PHT (%)	HT (%)	N	PHT (%)	HT (%)	N	PHT (%)	HT (%)	N	PHT (%)	HT (%)	N	PHT (%)	HT (%)
Thin	13	0.0	7.7	4	0.0	0.0	17	0.0	5.9	5	0.0	20.0	1	0.0	0.0	6	0.0	16.7
Normal	215	28.4	5.6	177	21.5	3.4	392	25.3	4.6	138	31.9	15.2	183	21.9	14.8	321	26.2	15.0
Overweight/obese	62	9.7	1.6	12	16.7	16.7	74	10.8	4.1	11	9.1	36.4	22	22.7	45.5	33	18.2	42.4
Total	290	23.1	4.8	193	20.7	4.1	483	22.2	4.6	154	29.2	16.9	206	21.8	18.0	360	25.0	17.5

PHT and HT stand for point-prehypertension and point-hypertension respectively

A total of 15.3% of the semi-urban population were overweight/obese, as against the 9.2% recorded in the urban area. Totally, 10.8% and 18.2% of those who were overweight/obese in the semi-urban and urban areas respectively had point-prehypertension. Also 4.1% and 42.4% respectively of the same group had point-hypertension. Both point-prehypertension and point-hypertension were more prevalent in the overweight/obese females compared to their male counterparts (Table 2).

**Figure 1:**
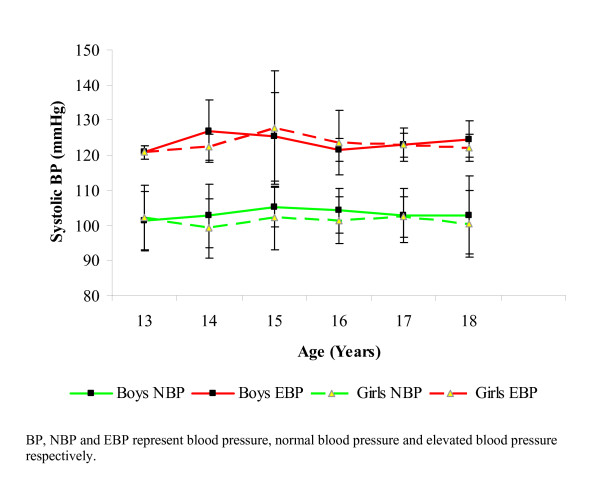
**Mean systolic blood pressures of subjects with normal and elevated blood pressures (NBP and EBP respectively)**. Differences between SBP means between the sexes for those with EBP and NBP (within the ages) were not significant (p > 0.05).

Mean systolic blood pressure was highest at 14 years (in boys) who had elevated blood pressures. The differences between the mean systolic blood pressures of the boys and the girls of the same age, who had elevated blood pressures was not significant (p > 0.05). Among the normotensive subjects, mean systolic blood pressure was highest at 15 years for boys and 17 years for girls. Normotensive males had a slightly and insignificantly (p > 0.05) higher mean systolic blood pressures compared to their female counterparts, in all the ages except at 13 years where the females had slightly higher mean systolic blood pressures than the males (Figure 1).

**Figure 2:**
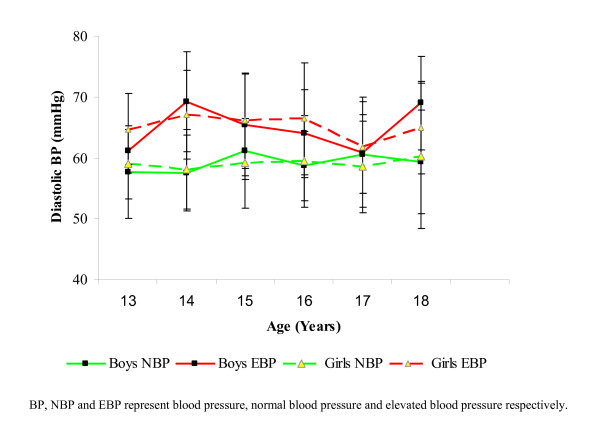
**Mean diastolic blood pressures of subjects with normal and elevated blood pressures (NBP and EBP respectively)**. Differences between DBP means between the sexes for those with EBP and NBP (within the ages) were not significant (p > 0.05).

The mean diastolic blood pressures, for both boys and girls of both blood pressure divides, all fell within the normal range. Mean diastolic blood pressure was highest for boys with elevated blood pressure at 14 years and 18 years, but at 14 years and 16 years for girls of the same category. In the subjects with elevated blood pressures, mean diastolic blood pressures was not significantly different (p > 0.05) between both sexes at any given age. For normotensive subjects, mean diastolic blood pressures peaked at 15 years and 17 years for boys and 18 years for girls (Figure 2). The differences between the mean diastolic blood pressures of male and female normotensives were not significant (p > 0.05) at any given age.

All the subjects reported being naïve to blood pressure measurement and none of those with elevated blood pressure was therefore aware of the condition.

## Discussion

Prehypertension is considered to be an indication of heightened risk for developing hypertension. Individuals in such category therefore need preventive health-related behaviors or therapeutic lifestyle changes [6]. Unfortunately, there is little or no data on adolescents with prehypertension especially in Nigeria. The 22.2% (23.1% for boys and 20.7% for girls) prevalence of point-prehypertension in the semi-urban area in this study; and the 25.0% (29.2% for boys and 21.8% for girls) prevalence for the same disorder in the urban area are quite high. The figures suggest that a large proportion of adolescents that were hitherto considered normotensive are actually at 'high risk' of developing hypertension. The increase in the proportion of adolescents with point-prehypertension from the semi-urban area to the urban area may be supportive of the claim that a shift in socio-economic gradient [that is usually correlated with an upward shift in geographic place of domicile (rural to urban)] often comes with lifestyle changes that predispose to chronic diseases [36,40].

The prevalence of point-hypertension in the study population, irrespective of place of domicile, is 10.1%. This is slightly higher than figures reported earlier in Nigeria [8,10]. It also shows a three-fold increase from figures reported three decades ago in Nigeria [9]. However, when separated based on place of domicile, it becomes clear that the increases have taken place mainly in the urban areas, as the prevalence of point-hypertension there was as high as 17.5% as against 4.8% in the semi-urban area. The prevalence of point-hypertension in the semi-urban area compares with figures from other (mostly developed) parts of the world [20,24,30,34]. This is worrisome and calls for urgent attention. However, what is more worrisome is that the prevalence of point-hypertension in the urban population is almost a 4-fold increase from the semi-urban group, and is one of the highest in recorded literature. The implications of such high rates on adult morbidity and mortality, and the burden it could place on health systems in Nigeria are palpable. These high rates of these disorders may be due to the rapidly changing lifestyles that come with urbanization - lifestyles that encourage unhealthy diets and habits, and reduced physical exertion, all of which predispose to chronic diseases [36].

As against point-prehypertension (in the urban area only), there was no significant sex difference in the prevalence of point-hypertension in both semi-urban and urban areas. This agrees with the reports of Chadha *et al *[16] and Prabhjot *et al *[7].

The prevalence of point-prehypertension increased almost 3-folds from the normal BMI-for-age group to the overweight/obese group only among the girls in the semi-urban area. The reverse was the case for boys in both places of domicile. The prevalence of point-hypertension increased almost 2-folds and 3-folds from the normal BMI-for-age group to the overweight/obese group among boys and girls, respectively in the urban area. In the semi-urban area, the increase was almost 5-folds in the same direction for girls but not boys. Point-prehypertension and point-hypertension increased with increasing BMI-for-age only in the girls of both localities while point-hypertension increased in the same vein only in urban boys. The relationship between blood pressure and BMI has been severally demonstrated in epidemiologic studies [14,17,28,29,36,41,42]. Hypertension in overweight/obese and overweight adolescents may arise from increased cardiac output, physical inactivity, excessive sodium intake and alteration in receptors for various presser substances [14].

The increase in mean systolic and diastolic blood pressures with increasing age is one of the most reproducible findings in the blood pressure epidemiologic literature. The differences in mean systolic and diastolic blood pressure values of both normotensive subjects and those with elevated blood pressures did not vary significantly between the sexes. This may be due to physiological and biochemical factors not accounted for in this work. The fact that the mean diastolic blood pressures for both boys and girls, who had elevated blood pressure or were normotensive, all fell within the normal range suggests a preponderance of systolic hypertension in the studied population. This is in agreement with some previous studies [27,43,44]. Mancia *et al *[45] earlier reported that systolic hypertension in children represents an early stage of essential hypertension that may indicate a hyperdynamic state which may be a pointer to basal sympathetic nervous system hyperactivity.

The fact that none of the subjects with elevated blood pressure was aware of the condition is an important finding. Dindar *et al *[25] had earlier reported similar findings. In fact, none of the subjects reported having had his/her blood pressure measured at any time, prior to this study. This corroborates the need for routine blood pressure monitoring in children and adolescents as that would help in the early initiation of management and by extension, reduce the burden of morbidity and mortality from hypertension and its sequelae.

Our study may be limited by the fact that what we report is 'point-hypertension' and 'point-prehypertension'. Hypertension can only reliably be established after multiple measurements on at least three different occasions. This deficiency could imply an over-estimation of the disorders in our study. We also used an oscillometric device as against the standard auscultation protocol. Oscillometric devices may not always match blood pressure values obtained by auscultation [46] but they have been shown to be valid for use in children and adolescents [42,47]. Our figures may even under-estimate the population with the disorders as automated devices are known to give lower blood pressure values than auscultation [48]. However, when viewed vis-à-vis the measurement of BP on a single visit, as we did, which may over-estimate the problems, our figures may be representative of the true situation in the studied population. We did not also assess factors like pubertal maturation, food choices, genetics, salt intake, etc, all of which may affect blood pressure elevation. Our small sample size especially at age 13 years in the urban area is due to non-cooperation by the students in that age range, while that of semi-urban 18 year-olds is due to the high rate of "school-drop-out syndrome" in the semi-urban area, as adolescents often drop out of school in order to earn a living and support their families, or as a result of marriage and pregnancy (for the girls). This precludes any objective interpretation of the data especially at those ages. Our study is representative of school-going adolescents in both localities. Extrapolating the findings to the general population (especially for other cultures) should be done cautiously.

## Conclusion

The prevalence of point-prehypertension and point-hypertension are considerably high in the studied populations. None of the affected subjects knew of the condition(s). Urgent public health action (like routine examination of BP of children and adolescents at each visit to the physician and before registration into secondary schools, the inclusion of hypertension-preventive health related behaviors in the Health Education curricula of schools and public enlightenment campaigns by government and public spirited individuals and organizations) is solicited, in order to fore-stall or attenuate the implications of adolescent hypertension especially as it tracks into adulthood.

## Competing interests

The authors declare that they have no competing interests.

## Authors' contributions

CECCE participated in the study design, analyzed and interpreted the data and wrote the manuscript. CEU conceived the study, participated in its design and coordinated data collection. LUSE participated in study design, and critically vetted the manuscript. All authors read and approved the final version of the manuscript.

## Pre-publication history

The pre-publication history for this paper can be accessed here:

http://www.biomedcentral.com/1471-2431/10/13/prepub
